# Bioinformatic Analysis Identified Hub Genes Associated with Heterocyclic Amines Induced Cytotoxicity of Peripheral Blood Mononuclear Cells

**DOI:** 10.3390/genes12121888

**Published:** 2021-11-25

**Authors:** Xinyang Li, Lu Dong, Huaning Yu, Yan Zhang, Shuo Wang

**Affiliations:** 1 Tianjin Key Laboratory of Food Science and Health, School of Medicine, Nankai University, Tianjin 300071, China; lixinyang@nankai.edu.cn (X.L.); donglu@nankai.edu.cn (L.D.); yzhang@nankai.edu.cn (Y.Z.); 2 Midea Group Guangdong Midea Kitchen Appliances Manufacturing Co., Ltd., Foshan 528000, China; yuhuaning1981@126.com

**Keywords:** PhIP, IQ, PBMC, WGCNA, hub gene

## Abstract

Heterocyclic amines (HCAs) are a set of food contaminants that may exert a cytotoxic effect on human peripheral blood mononuclear cells (PBMC). However, the genetic mechanism underlying the cytotoxicity of HCAs on PBMC has not been investigated. In the study, bioinformatic analysis on gene dataset GSE19078 was performed. The results of weighted correlation network analysis and linear models for microarray and RNA-seq data analysis showed that four gene modules were relevant to 2-amino-1-methyl-6-phenylimidazo[4,5-b]pyridine (PhIP) exposure while one gene module was correlated with 2-amino-3-methyl-3H-imidazo[4,5f]quinoline (IQ) exposure. Gene functional analysis showed that the five modules were annotated mainly with mRNA transcriptional regulation, mitochondrial function, RNA catabolic process, protein targeting, and immune function. Five genes, MIER1, NDUFA4, MLL3, CD53 and CSF3 were recognized as the feature genes for each hub gene network of the corresponding gene module, and the expression of feature genes was observed with a significant difference between the PhIP/IQ samples and the other samples. Our results provide novel genes and promising mechanisms for exploration on the genetic mechanism of HCAs on PBMC.

## 1. Introduction

Heterocyclic amines (HCAs) are potential mutagens ubiquitously existing in cooked protein-rich food such as fried fish and roasted meat [1]. Hiding behind the tempting flavor and rich nutrients of grilled food, HCAs are inevitably consumed by and absorbed into human bodies and exert toxicological effects [2]. Studies have found several kinds of HCAs. Among the types of discovered HCAs, 2-amino-1-methyl-6-phenylimidazo[4,5-b]pyridine (PhIP) and 2-amino-3-methyl-3H-imidazo[4,5f]quinoline (IQ) are the most common HCAs, and are classified as group 2B and group 2A carcinogens respectively by the International Agency of Research on Cancer (IARC) [3]. As carcinogenic xenobiotics consumed with diet and absorbed by the digestive tract, HCAs have been found to exert their carcinogenic effect in the colon [4]. Subsequent studies found that breast and prostate are also target organs of HCAs [5,6]. The potential mechanism underlying the carcinogenicity of HCAs includes oxidative stress, DNA damage, endocrine disruption, etc. [7]. However, carcinogenic toxicants are usually implicated in immunosuppression [8]. So far, only a few studies have focused on the immunotoxicology of HCAs and their cytotoxic effect on immune cells, and little information is known about the underlying mechanism of cyotoxicology of HCAs on immune cells. A recent study investigated the genotoxicity of HCAs on isolated human peripheral blood mononuclear cells (PBMC), suggesting a DNA damage effect of HCAs on PBMC [9]. However, the other mechanisms, especially the genetic mechanism, behind the cytotoxic effect of HCAs on PBMC has not been elucidated.

Bioinformatic analysis is the computational approach for analyzing complex genetic profiles and is essential for disentangling the mechanisms underlying cellular development, disease progression, and other biological and pathological processes [10]. Bioinformatic analysis has played a pivotal role in cancer research and shown unique advantages compared with experimental cancer research with respect to the 3Rs for animals with replacement, reduction and refinement [11]. As a novel approach with a large advantage in biomedical research, more and more toxicological studies have employed bioinformatic analysis as a novel method exploring the toxicology, especially in the terms of the toxicological mechanism. The authors have successfully used bioinformatic analysis in the exploration of mechanism toxicology of PhIP and other carcinogens [12,13], and found interesting changes in colonic transcriptome induced by PhIP [12], suggesting that bioinformatic analysis might be a good utility for exploring the toxicological mechanism of food contaminants. In the last ten years, more and more novel methods of bioinformatic analysis, such as weighted gene correlation network analysis (WGCNA) and linear models for microarray and RNA-seq data (LIMMA) analysis, have been established to identify the potential signaling pathways and plausible central modulating genes (hub genes) [14] in biomedical research.

In the study, two bioinformatic methods: WGCNA [15,16] and LIMMA [17], were employed to integratively analyze the microarray GSE19078 from the Gene Expression Omnibus (GEO) database. After focusing on PhIP and IQ dosed groups and compared with other groups, five sets of hub genes and five feature genes modulating PBMC progression in response to PhIP or IQ exposure were identified, and several pathways were found to get involved in the cytotoxic effect of PhIP and IQ on PBMC. The present study provides promising genes and plausible mechanisms underlying the toxicity of HCAs on PBMC.

## 2. Materials and Methods

### 2.1. GEO Data Accession

GEO (http://www.ncbi.nlm.nih.gov/geo/, accessed on 18 November 2021) is a public database providing functional genomic information from high-throughput gene expression data. A search of HCA and PBMC was conducted in the GEO database with the following keywords: (“PhIP AND IQ” and “peripheral blood mononuclear cells”). Only one dataset GSE19078 was found. GSE19078 was a dataset initially performed in 2009 to explore the transcriptomic profile of PBMC with immunotoxic exposure from eight different immunotoxicants and four non-immunotoxic xenobiotics, including PhIP and IQ, served as the control, in which the authors did not investigate the effect of PhIP or IQ [18]. The GEO dataset was downloaded and imported into R (https://www.r-project.org/, accessed on 18 November 2021, version 4.0.2) by using R package “GEOquery” version 2.58.0 [19].

### 2.2. WGCNA Analysis

Weighted gene co-expression network analysis (WGCNA) was performed by using R package “WGCNA” (version 1.70-3) [15,16] to explore traits-related modules. Considering that WGCNA quantifies the module-trait association by calculating the Pearson correlation and the trait variable should be numeric, the traits were assigned with values of 1 or 0 based on whether the sample was exposed with a specific xenobiotic or not. The data matrix consisting of all 89 samples was transformed into a topological overlap matrix (TOM). In the present work, we set soft-thresholding power at 8, scale free R-square at >0.80 and minimal module size at 30 to figure out key modules. The verbose value of all relative functions was set at three. The correlation between gene module and traits were set as correlation coefficient *r* > 0.40 and significance *p* < 0.05, in which *r* of 0.40 was generally recognized as a threshold between no/very weak correlation and weak correlation.

### 2.3. LIMMA Analysis

To study the differential expression of genes between the PhIP/IQ group and other groups (as the control), the LIMMA analysis was performed by using R package “LIMMA” (version 3.46.0). The adjusted *p-*value < 0.05 were defined as the thresholds for the screening of differential expression of genes. Probes without corresponding gene symbols from the annotation and genes excluded by ‘goodSamplesGenes’ in WGCNA were excluded from the LIMMA analysis.

### 2.4. Functional Analysis

To further explore the function of genes in different modules, the gene lists of differentially expressed genes and key modules were analyzed by functional enrichment. Gene Ontology (GO) is a widely-used tool for annotating genes with functions, especially molecular function, biological pathways, and cellular components [20,21]. Kyoto Encyclopedia of Genes and Genomes (KEGG) Enrichment Analysis is a practical resource for analytical study of gene functions and associated high-level genome functional information (https://www.kegg.jp/, accessed on 18 November 2021). The ClusterProfiler package (version 3.18.1) in R was employed to analyze the GO function of potential targets and enrich the KEGG pathway [22]. The *p-*value < 0.05 was set as the inclusion criterion for pathways.

### 2.5. Network Construction and Hub Gene Identification

The table file containing node genes and inter-node weight values generated by WGCNA for the network, was analysed by visANT (http://visant.bu.edu/, accessed on 18 November 2021). By gradually narrowing down the range of weight cutoff, a reasonable number (10–50) of hub genes of target modules from WGCNA analysis were identified, and a “feature gene” of a set of hub genes were identified with high gene significance, high module membership and significant difference in gene expression as measured in LIMMA analysis. 

### 2.6. Statistical Analysis and Plotting

A Wilcoxon test was performed on R for comparing the expression of feature genes between the PhIP/IQ group and the control, and a *p-*value < 0.05 was considered statistically significant. Boxplots of gene expression were plotted by R. A Venn plot was performed to collect the consensus genes from different gene sets by using Venny 2.1.0 [23].

## 3. Results

### 3.1. Weighted Gene Coexpression Network Analysis

**Figure 1:**
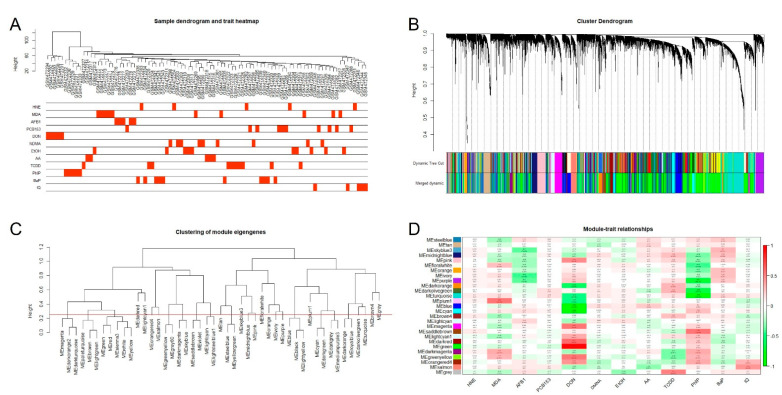
The WGCNA result of GSE19078. (**A**) The sample dendrogram and trait heatmap of GSE19078 (n sample = 89). The clustering dendrogram of samples was based on their Euclidean distance. (**B**) Identification of gene coexpression modules in GSE19078. (**A**) Clustering dendrogram of genes, with dissimilarity based on topological overlap, together with assigned merged module colors and the original module colors. (**B**) The Dynamic Tree Cut of original module colors. (**C**) To merge modules, the height cut was set at 0.25, corresponding to the correlation of 0.75. (**D**) The module-trait associations. Each row corresponds to a module eigengene, and column to a trait (treatment). Each cell contains the corresponding correlation Pearson *r* and *p-*value. The table is color-coded by the correlation according to the color. The correlation Pearson *r* value and the significance between gene modules and the clinical traits are available in Supplementary Table S2 and Supplementary Table S3, respectively. The full names for abbreviations of the 12 chemicals is available in Supplementary Table S4.

The GEO dataset GSE19078 contains 41,000 features from 89 samples. After checking the data with missing entries and ruling them out, 17,970 genes (Table S1) from 41,000 features were identified and the following weighted gene network construction was pursued. By performing hierarchical cluster analysis, all 89 samples were included, and no outlining sample was found (Figure 1A). After relating the cluster dendrogram with treatment (traits data), we found that all five of the PhIP group samples were in the same cluster, while four out of five IQ group samples were in the same cluster (Figure 1A). These results show a good quality of the data and a similarity among the expression profile within the IQ dosed group or the PhIP group.

After clustering the samples, we performed the network construction and module detection. To construct a weighted gene network entails the choice of the soft thresholding power *β*, to which co-expression similarity is raised to calculate adjacency [15]. After proceeding with the scale-free topology, we choose the power 8, which is the lowest power for which the scale-free topology fit index reaches 0.80 (Figure S1). We then use power 8 to calculate the adjacency and the topological overlap matrix (TOM), resulting in a clustering dendrogram of a gene (Figure 1B,C) with 47 gene modules. After merging these modules with a hightcut threshold 0.25 (Figure 1C), a merged dendrogram was produced (Figure 1B) in which all 17,970 genes were clustered into 26 modules (the grey colored module contained the genes unclustered). The name of module colors and the number of genes are shown in (Figure S2).

**Figure 2:**
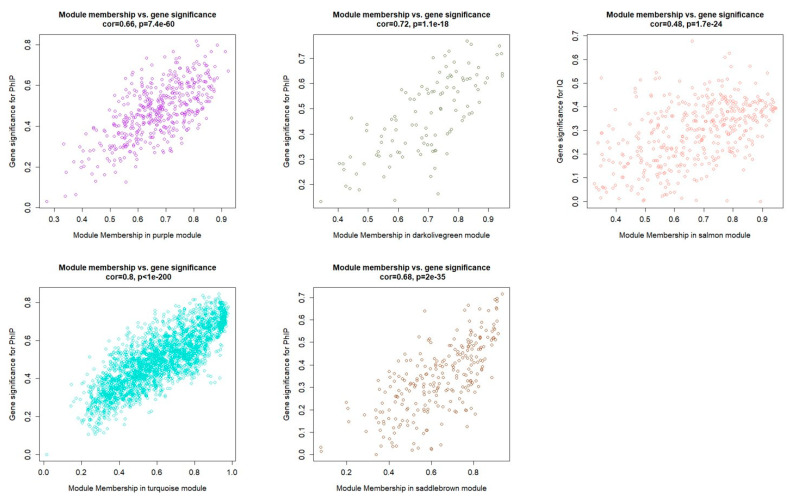
The scatterplot of gene significance for weight vs. module membership in the purple, darkolivegreen, turquoise, saddlebrown, and salmon modules. The correlation was calculated with the absolute value of gene significance and module membership, which illustrates the association between genes and the trait.

Finally, we related the gene modules with treatment traits to identify important genes. After performing the module-trait association analysis (Figure 1D, values are available in Tables S2 and S3), we set correlation absolute value > 0.40 and *p*-value < 0.05 as the threshold for significant modules. Only the salmon module was found to be correlated with IQ, while four gene modules: turquoise, darkolivegreen, saddlebrown, and purple were found to be significantly correlated with PhIP (Figure 2).

### 3.2. Functional Analysis of the Correlated Gene Moudules

**Figure 3:**
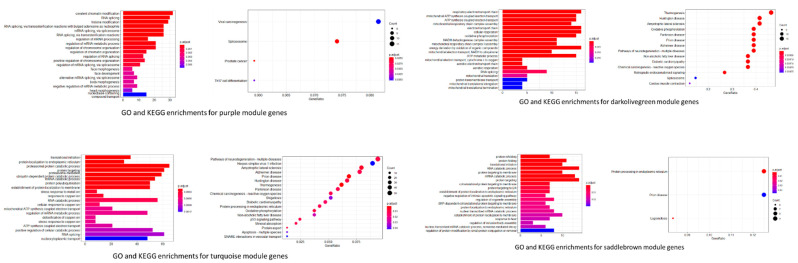

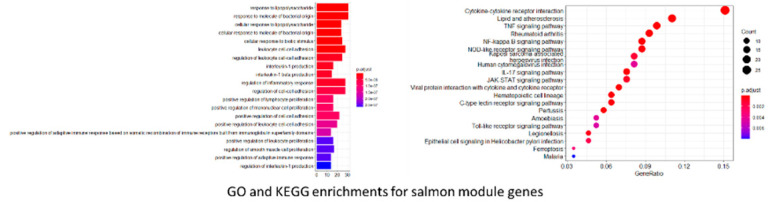
The GO and KEGG enrichment analysis for the genes in modules. The module genes of purple, darkolivegreen, turquoise, saddlebrown, and salmon modules were analyzed and the top 20 of the enrichment GO-term and KEGG-pathway based on the adjusted *p*-value were shown in the figures in the order of *p*-values.

To explore the function of the correlated gene modules, GO and KEGG enrichment analyses were performed on correlated gene modules. As is shown in Figure 3, genes from the five relevant gene modules were enriched in different gene functions. The four PhIP relevant modules: purple, darkolivegreen, turquoise and saddlebrown, were annotated mainly with mRNA transcriptional regulation, mitocondral function, RNA catabolic process, protein targeting, and immune function. The salmon module, which is the only IQ relevant module, was annotated mainly with immunological cell response.

### 3.3. The Identification of Differentially Expressed Genes 

**Figure 4:**
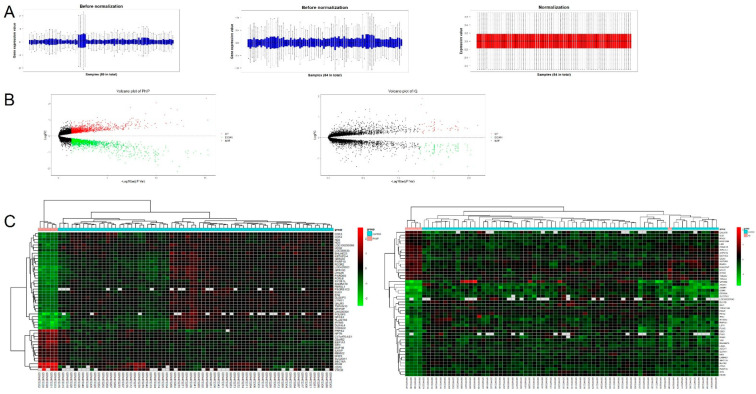
The LIMMA analysis for differentially expressed genes (DEGs) in the PhIP and IQ group. (**A**) The gene expression values of samples in GSE19078. Five samples, “GSM472290” “GSM472291” “GSM472292” “GSM472293” “GSM472294” (deoxynivalenol group) excluded for their high values may not be suitable for linear module analysis. (**B**) the volcano plot of PhIP and IQ, with the adjusted *p*-value < 0.05 as the thresholds for DEGs. (**C**) The top 50 most differentially expressed genes in the PhIP and IQ group, calculated with the normalized expression value of each gene.

By performing LIMMA analysis, the differentially expressed genes (DEGs) of PhIP and IQ groups were identified. After ruling out probes with too many missing values and probes without matched gene IDs, 10,594 genes (Table S5) were found suitable for LIMMA analysis. From the boxplot of gene expression values of all 89 samples, we found that sample “GSM472290” “GSM472291” “GSM472292’ “GSM472293” “GSM472294” were different from other samples (Figure 4A), which were then dosed with deoxynivalenol. Since the study does not focus on the toxic effect of deoxynivalenol, we exclude the deoxynivalenol group to avoid potential influence of extreme values, and the remaining 84 samples were enrolled for LIMMA analysis after normalization (Figure 4A). By setting the threshold with adjusted *p-*value < 0.05, the result showed 2620 DEGs of PhIP with 1318 down-regulated and 1292 up-regulated, and 145 DEGs of IQ with 88 down-regulated and 57 up-regulated (Figure 4B, Tables S6 and S7). The top 50 most differentially expressed genes for PhIP or IQ were shown in Figure 4C, respectively. Also, we performed GO and KEGG enrichment analysis on DEGs of PhIP or IQ groups, and the result also suggests that transcriptional regulation and mitochondrial function were the plausible signaling pathway of PhIP, while immune progress was the probable signaling pathway of IQ, which is shown in Figure S3.

### 3.4. Hub Gene Identification

**Figure 5:**
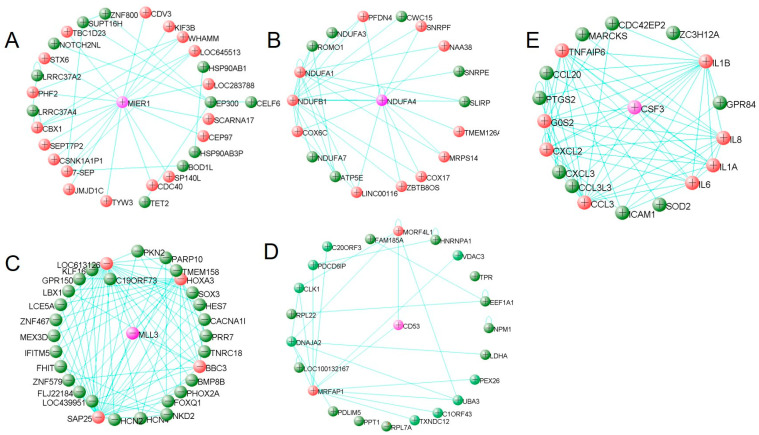
The hub genes and feature genes from each of the five modules. (**A**) 30 hub genes identified as strongly connected in purple module with weight cutoff at 0.08. (**B**) 19 hub genes identified as strongly connected in darkolivegreen module with weight cutoff at 0.08. (**C**) 35 hub genes identified as strongly connected in turquoise module with weight cutoff at 0.41. (**D**) 21 hub genes identified as strongly connected in saddlebrown module with weight cutoff at 0.09. (**E**) 19 hub genes identified as strongly connected in salmon module with weight cutoff at 0.20. Among these hub genes, the feature gene was colored purple and the hub genes directly correlated with the feature gene at the current weight cutoff value were colored red.

**Figure 6:**
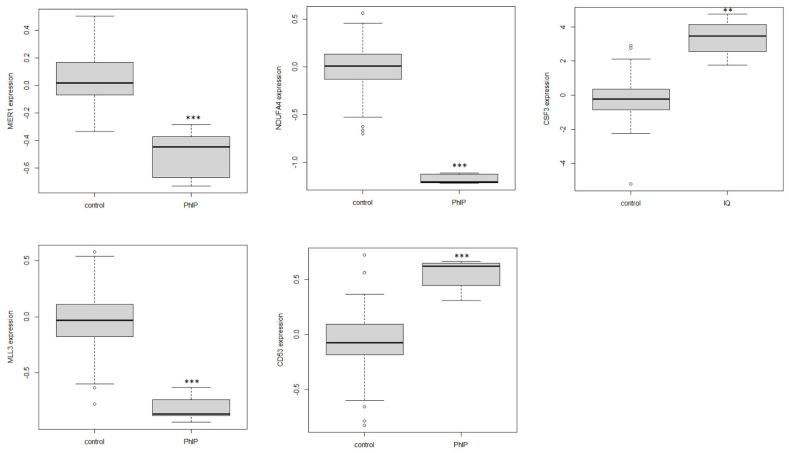
The gene expression of five feature genes between dose and control group. The gene expression values from 84 samples after normalization by LIMMA analysis was used to plot the boxplot; The value of genes in PhIP or IQ samples were the dose group and the other 79 samples served as the control. A Wilcoxon test was employed to compare the difference between the dose group and the control, with ** *p*-value < 0.01 and *** *p*-value < 0.001.

By using visANT to identify high module membership genes, a number of hub genes that were selected for each related module are presented in Figure 5. Moreover, by shifting the layout of hub genes at the current weight cutoff level, five genes, MIER1, NDUFA4, MLL3, CD53 and CSF3, were recognized as the feature genes for each hub gene network from the purple, darkolivegreen, turquoise, saddlebrown and salmon modules, respectively. Finally, the expression of the 5 feature genes was verified by comparing the expression level between the PhIP/IQ group and the control, and the boxplot in Figure 6 shows that the expressions of the five feature genes are significantly different between PhIP/IQ groups and the control.

## 4. Discussion

In the present study, we investigated the genetic mechanism underlying the cytotoxic effect of HCAs on PBMC by analyzing the GEO database GSE19078 through bioinformatic methods. Five gene modules were identified that are relevant with PhIP/IQ exposure, whose gene mainly annotated with mRNA transcriptional regulation, mitochondrial function, RNA catabolic process, protein targeting, and immune function. For each module, a set of hub genes was identified, and five feature genes, which were most connected with other hub genes and therefore considered as the “significant gene”, including MIER1, NDUFA4, MLL3, CD53 and CSF3, were characterized. Our study exhibited genetic evidence for the cytotoxic effect of HCAs on PBMC, providing novel clues for investigating the mechanism of the immunotoxic effect of HCAs. 

In the study, five groups of hub genes were identified and five feature genes were selected and served as the most connected genes for the 5 modules. All the 5 genes, MIER1, NDUFA4, MLL3, CD53 and CSF3, showed significant differences between PhIP/IQ group and the control (Figure 6), indicating a good representative role of the five genes in modulating genetic function under PhIP/IQ treatment. MIER1 is a transcriptional regulator acting in the mesoderm induction early response [24]. One of the isoform of MIER1, the MIER1-alpha, has been reported as a novel estrogen receptor binding protein [24,25]. Meanwhile, one of the carcinogenic mechanisms of PhIP is that it may interact with the estrogen receptor and thus promote breast cancer [26,27]. NDUFA4, also named as COXFA4, is a cytochrome-c oxidase subunit. By interacting with other cytochrome-c oxidase proteins, such as NDUFA1, NDUFB1 and COX6C etc., NDUFA4 participates in the cytochrome-c oxidase activity [28]. It is in accordance with a recent study that PhIP could induce cytotoxicity of liver cells through cytochrome-c mediated oxidative stress [29]. MLL3 and the connected hub genes in the saddlebrown module, including HOXA and SAP25 (Figure 5), are all genes that participated in the RNA synthesis and transcriptional complex [30]. While it has been reported that 3-Amino-1,4-dimethyl-5H-pyrido[4,3-b]indole, another kind of HCA, may modulate lipopolysaccharide-induced interleukin-8 expression by decreasing mRNA stability [31]. This study and our results both suggest that HCAs may regulate RNA synthesis, transcription, and stability, and may therefore exert cytotoxic effects. CD53 and CSF3 are both immune-regulating genes [32,33]. The gene enrichment result of both WGCNA and LIMMA analysis both showed that immune function was the target pathway of PhIP or IQ exposure (Figure 3 and Figure S3). Above all, although not being researched by other studies previously, the five feature genes, MIER1, NDUFA4, MLL3, CD53 and CSF3, likely participate in the cytotoxic effect of HCAs, to which further research on the mechanism of HCAs toxicity should be directed.

Immunotoxicity is an important part of the toxic effect of xenobiotics, and has been considered one of the carcinogenic mechanisms of food and environmental carcinogens [34]. As the confirmed human carcinogen in food, the toxicological effect of HCAs on immune cells has been investigated for decades, but few studies have focused on the mechanism. In the previous study which provided the dataset GSE19078, PhIP and IQ did not show an immunotoxic effect on PBMC at the designated treatment, and were therefore taken as non-immunotoxic chemicals and served as the control [18]. A study afterwards also took HCAs as the non-immunotoxic agents [35]. However, our result showed that both PhIP and IQ may affect immune-regulating genes and pathways (Figure 3 and Figure S3). As a matter of fact, some HCAs have been revealed with an influence on immune functions. As early as 1994, PhIP has been evaluated with immunotoxicity and was found with a depletion of T cells and a slight increase in B cells in mice with oral administration of PhIP [36]. Apart from T cells and B cells, the following studies also found that PhIP may suppress tumor necrosis factor-alpha in mice immune RAW 264.7 cells [8]. Another kind of HCA, 3-Amino-1,4-dimethyl-5H-pyrido[4,3-b]indole, was found to modulate lipopolysaccharide-induced interleukin-8 expression on immune THP-1 cells [31]. All these studies suggest that HCAs may affect immune cell proliferation and cell function. In the present study, our results showed that several inmmune-modulating genes, including CD53 and CSF3, that were involved in cell response to HCAs treatment may be promising mechanisms for further research on HCAs-induced cytotoxicity on PBMC cells. In recent years, more and more novel methods have been applied in toxicology research. New methods have been emerging in the field of descriptive toxicology [37] and mechanistic toxicology [38]. Bioinformatic analysis is a battery of techniques that use computerized systems to analyze large bodies of biological data and find possible genetic relationships in various biological processes. Bioinformatics has been largely applied in biological research, pathological research, and cancer research, and was regarded as a novel approach to exploring the cancer mechanism with respect to the 3Rs for replacement, reduction and refinement [11]. Recently, a growing number of toxicological studies has employed bioinformatics as a new method [39], showing great advantage on providing genetic explanations of the experimental toxicity [40]. Our previous studies have successfully used bioinformatic methods to explore genes in amino acid metabolism and fecal metabolites, as well as bacterial composition [12], showing a wider range of utility of bioinformatic in the research of food toxicology than other toxicology fields. However, in general, the gene datasets on toxicology were still much less than in other fields, e.g., cancer research. During the research for the study, we searched on the GEO database website and tried to include all HCA-related datasets. However, only one dataset, GSE19078, was found and included for bioinformatic analysis. As a matter of fact, this data was not only the lone dataset referring to the toxic effect of HCAs on PBMC, but also the only dataset related with HCAs exposure. In short, we think that the application of bioinformatic analysis in toxicology has its unique advantage, especially in the field of food toxicology, and more genomic data regarding toxicology is in great demand.

WGCNA is an analytical method for clustering gene modules of highly correlated genes, which are then related to the modules with clinical traits [16]. In the current study, not only the modules relevant with PhIP or IQ treatment could be explored, but the gene modules with other traits, such as MDA (malondialdehyde), could also provide interesting gene modules for future studies on MDA (Figure 1D). Besides, samples from DON (deoxynivalenol) groups showed a distinct branch from other groups in the dengram from hierarchical clustering (Figure 1A), and a much higher connection with gene modules (Figure 1D) in WGCNA than other toxicants, and all the samples treated with DON showed a distinctive feature as measured by LIMMA analysis (Figure 4A). Therefore, five samples from the DON group were not included in the control when obtaining the DEGs of PhIP/IQ by LIMMA analysis. Meanwhile, these results suggest that the genetic change of DON on PBMC may be different from the other 11 kinds of chemicals, which may be of interest for future studies on the toxic effect of DON. Meanwhile, our study also suggests that genome technology and bioinformatic analysis are good utilities in simultaneously investigating the genetic changes induced by multiple toxicants.

In the current study, we chose a correlation coefficient *r* > 0.40 as the threshold to identify trait-correlated gene modules. Although 0.40 is a commonly used cutoff value for correlation coefficient between no/very weak correlation and weak correlation, it is still worth noting that if we increase the *r* value, for example, up to 0.50, there would be no modules correlated with IQ treatment. And the salmon module, which was annotated mainly with immunological cell response (Figure 3), would not be correlated with IQ, and no correlated gene module for IQ would be found. However, our results of LIMMA analysis showed that the DEGs between IQ and the control were enriched in pathways of immune cell response. And the feature genes for salmon module CSF3, were also inmmune-regulating genes, indicating an immunotoxic effect of IQ [33]. This reminds us that the cutoff of correlation coefficient (*r* value) should be carefully set when performing WGCNA. And for bioinformatic analysis, multiple methods should be performed to verify the result from only one method.

visANT is an open access software for analyzing networks in cells. Compared with other network analyzing platforms such as STRING (https://www.string-db.org/, accessed on 18 November 2021), visANT has its advantage of taking the connection weight between genes into analysis, and connecting genes based on the calculated result but not the signaling pathways that exist. By using visANT or Cytoscape (https://cytoscape.org/, accessed on 18 November 2021), as recommended by WGCNA [15,16], our result can reflect the connection based on the weight value from WGCNA results, but not from the connection from existing pathways, and hub genes, the most connected genes or the core-regulator genes, were found and represented in our result (Figure 5). By shifting the layout of hub genes in visANT, a feature gene was posed at the centre of the hub genes. Actually, hub genes and feature genes are new concepts with the meaning of genes with high correlation in candidate modules. There are multiple methods for selecting the hub genes and feature genes and the methods and results might be different. In our study, we used viaANT to identify a reasonable number (10–50) of genes that connect with each other at the designated connection weight, and one central gene for each set of hub genes was selected as the feature gene for expression verification. The expression of all the five feature genes were significantly different between PhIP/IQ and the control groups (Figure 6), showing a good representative role of the five genes. However, the other hub genes (Figure 5), in combination with the mechanisms shown in gene enrichment analysis (Figure 3 and Figure S3), are still worthy of exploration in future studies in order to better understand the genetic mechanism of HCAs toxicity.

## 5. Conclusions

In conclusion, we identified five gene modules and their hub genes related with HCAs-induced PBMC cytotoxicity. We found that MIER1, NDUFA4, MLL3, and CD53 are the possible feature genes for PhIP exposure, while CSF3 is the feature gene for IQ exposure. Cytochrome-c mediated oxidative stress, RNA synthesis dysregulation and immune dysfunction are probably the genetic mechanism for HCAs-induced cytotoxicity in PBMC. Experimental research in future studies is needed to further verify and explore the mechanisms of HCAs toxicity.

## Data Availability

The array data GSE19078 included in the study are openly available in Gene Expression Omnibus at https://www.ncbi.nlm.nih.gov/geo/query/acc.cgi?acc=GSE19078 (accessed on 18 November 2021), reference number [18].

## Supplementary Materials

The following are available online at https://www.mdpi.com/article/10.3390/genes12121888/s1, Table S1:The gene symbols and the module colors of all 17,970 genes, Table S2: The correlation Pearson *r* value between gene mod-ules and the clinical traits (treatment), Table S3: The *p* value of the correlation Pearson *r* value between gene modules and the clinical traits (treatment), Table S4: The abbreviations and full names of 12 chemicals, Table, S5: All the 10594 genes included in LIMMA analysis, Table S6: The differentially expressed genes (DEGs) of PhIP group, Table S7: The differentially expressed genes (DEGs) of IQ group, Figure S1: Analysis of network topology for various soft-thresholding powers, Figure S2: The name of module colors and the number of genes within, Figure S3: The GO and KEGG enrichment analysis for DEGs of PhIP and IQ. Computer Code are available at: http://xinyang221.gitee.io/blog/2021/10/18/supple.%20mdpi-gene-20211018/ (accessed on 18 November 2021)
