# Supplementary figures and images for: Bioinformatic Analysis Identified Hub Genes Associated with Heterocyclic Amines Induced Cytotoxicity of Peripheral Blood Mononuclear Cells
Source: Genes (Basel). 2021 Nov 25;12(12):1888. doi: 10.3390/genes12121888 (PMC8700875; doi:10.3390/genes12121888)

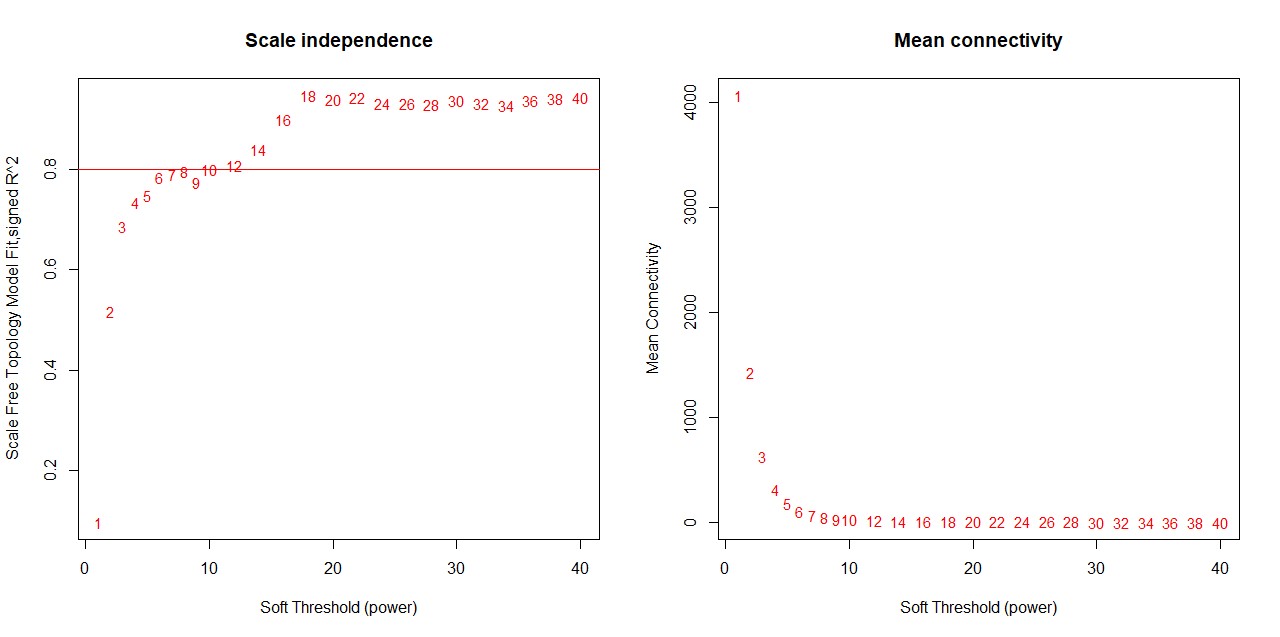

Supplement: Supplementary file 1 [file genes-12-01888-s001.zip › supplementary data 2021-11-25/Figure S1.jpg]

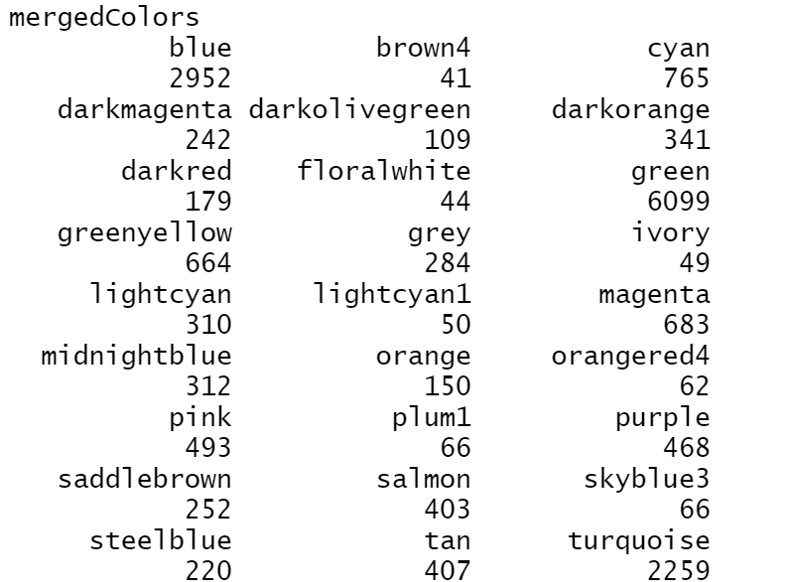

Supplement: Supplementary file 1 [file genes-12-01888-s001.zip › supplementary data 2021-11-25/Figure S2.jpg]

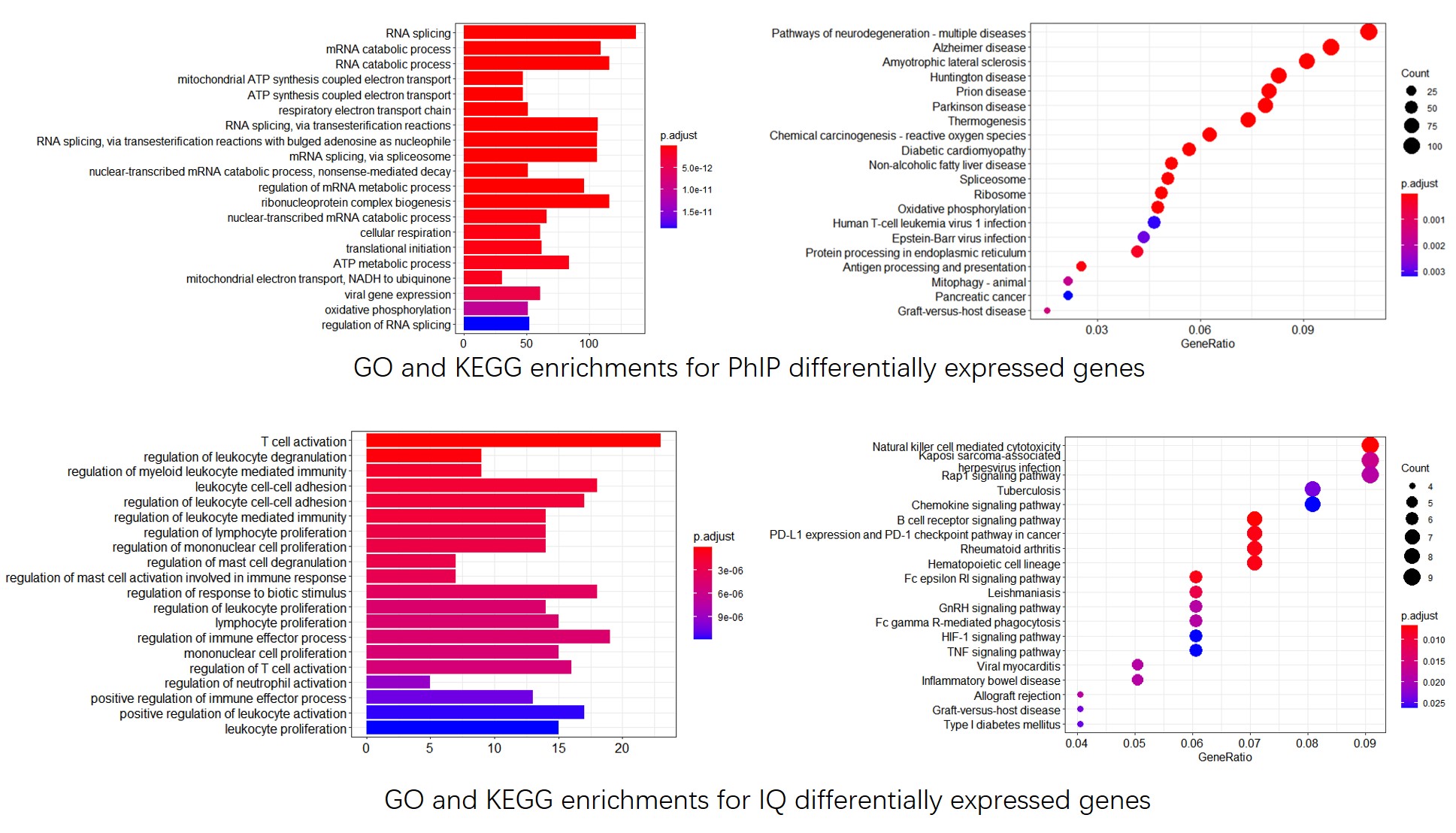

Supplement: Supplementary file 1 [file genes-12-01888-s001.zip › supplementary data 2021-11-25/Figure S3.jpg]
